# The usability of virtual reality to train individuals in responding to behaviors related to dementia

**DOI:** 10.3389/frdem.2023.1237127

**Published:** 2024-01-08

**Authors:** Linda Garcia, Annie Robitaille, Stéphane Bouchard, Marie-Christine Rivard, Lynn McCleary

**Affiliations:** ^1^Interdisciplinary School of Health Sciences, University of Ottawa, Ottawa, ON, Canada; ^2^Department of Psychoeducation and Psychology, University of Quebec in Outaouais, Gatineau, QC, Canada; ^3^Department of Nursing, Brock University, St. Catharines, ON, Canada

**Keywords:** aging, long-term care home, health care, virtual humans, aged care, responsive behavior, training

## Abstract

**Introduction:**

Dementia is associated with several behavioral changes globally referred to as *Behavioral and Psychological Symptoms of Dementia* (BPSD) of which many are recognized to be the expression of unmet needs triggered by environmental factors. BPSD are an extreme source of stress for family care partners and health care providers alike and can be the reason why people living with dementia (PLWD) are placed in long-term care homes (LTCH). The overall goal of this project was to examine whether a virtual environment that includes a virtual LTCH resident with dementia in a lifelike situation could be useful and usable for health care providers and care partners to identify potential triggers to BPSDs while being engaged emotionally with the scenario.

**Methods:**

Twenty-three health care professionals working with PLWD, 25 care partners to PLWD, 27 students in a health-related field, and 11 university/community college faculty members teaching courses relevant to gerontology tested the application which depicted a meal-time scenario. In addition to being asked about the behavioral triggers in the scene, participants were asked about the usefulness and usability of the tool for training. Presence and simulator sickness were also measured.

**Results:**

Results suggest that participants generally felt present and emotionally engaged. They could identify the potential triggers for the observed behaviors in the virtual human with dementia as well as suggest some solutions. The majority (87% of participants) found the tool easy to use. Many participants identified the inability to interact with the virtual humans as a shortfall, and few reported mild to moderate levels of simulator sickness.

**Discussion:**

As the behavioral changes associated with dementia can cause extreme stress for those interacting with PLWD, developing an effective and efficient training tool could significantly improve well-being for all involved. The investigators see the development and testing of an interactive version of this virtual environment as a next step in making this a clinically relevant training tool.

## 1 Introduction

According to recent estimates, about 50 million people worldwide are currently living with dementia, with the figure projected to more than triple by 2050 (World Health Organization, 2017). For this reason, enhanced understanding and support related to dementia care have been noted as key global public health priorities (World Health Organization, 2012). In addition to the numerous symptoms related to dementia such as memory loss and difficulty performing familiar tasks, dementia is associated with a number of behavioral changes globally referred to as Behavioral and Psychological Symptoms of Dementia (BPSD; Cloak and Al Khalili, 2022). Full comprehension of the nature and etiology of the symptoms associated with these behaviors continues to be the subject of much discussion. For the purposes of this paper, the term BPSD will be used, recognizing that they do not form a homogenous grouping of symptoms (van der Linde et al., 2014), and most importantly, that many of these behaviors are the result of the expression of unmet needs as a response to social and physical environmental triggers (Dupuis et al., 2012; Warren, 2022) that can change over time (Robitaille et al., 2023). BPSD are a source of stress for care partners and are sometimes the reason why families feel they can no longer meet the needs of their loved ones, hoping that long-term care homes (LTCH) are better equipped to manage the occurrence of these behaviors. Despite the existence of programs targeting future and existing health care providers and families in their development of strategies to reduce the triggers to BPSD, they continue to be a source of concern and interest.

Living with or serving as care partners to people with advanced dementia can certainly be challenging. The stress some families experience becomes overwhelming as they juggle their many responsibilities and see the person they once knew react in ways that are foreign to them. As options diminish for living at home, health care providers in collective dwellings such as LTCHs become part of the circle of care. Given that this is a novel environment for the person living with dementia (PLWD), health care providers might find these residents react in more intense ways as they try to make sense of their social and physical environments. Health care providers committed to offering person-centered care must therefore understand what those triggers might be. Hence, understanding the relationship between the resident's behaviors and the possible triggers in the environment is paramount to helping both the PLWD and those around them improve their quality of life.

While not all behavioral symptoms can be attributed to changes in environment, it is now well-recognized that non-pharmacological interventions can be effective and are recommended before attempting to manage the behaviors with pharmaceuticals (Caspar et al., 2018). Removing the triggers of behaviors will reduce the risk of undo side effects of medications as well as help prevent further BPSD. There is therefore a need to further bring awareness to health care providers and care partners that their behaviors, along with the physical environment, can serve as triggers that can be reduced through the use of appropriate strategies.

A few programs have successfully used simulated patients (actors) to coach family care partners in practicing new approaches when interacting with loved ones who have dementia (Chiu, 2013; Sadavoy et al., 2021). In some sectors, these programs can be expensive to run as they require individuals to participate in a series of weekly role-playing sessions, offered at specific times and locations based on the availability of the instructors and participants. Given care partners' and health care providers' other time commitments, attending these sessions can be challenging. While better than classic classroom learning, it may also be difficult, in these role-playing sessions, to replicate the true overwhelming emotions that often occur when faced with these behaviors. Real life may elicit more visceral emotional reactions that cause the learner to forget what they need to do.

One option that might elicit closer-to-reality emotions is the use of technology such as immersive virtual reality (IVR). IVR can be described as “the use of computer and behavioral interfaces to simulate the behavior of 3D entities that interact in real time with each other and with a user immersed via sensorimotor channels” (Bouchard and Rizzo, 2019). It has been shown to help bridge the gap between clinic-based techniques and real-world applications to anxiety-provoking situations (Maples-Keller et al., 2017; Carl et al., 2019). Research has shown time and again that creating controlled anxiety-provoking virtual environments are not only possible, but effective. IVR has successfully been used to treat anxiety disorders and to train medical students by adding psychological realism to the intervention (Maples-Keller et al., 2017; Carl et al., 2019; Javaid and Haleem, 2020).

While there are many great examples starting in the early 90's of how patients could be treated for anxiety by immersing them in virtual environments, few, partly because of technological advances and cost of equipment, had created interactive scenarios with virtual humans; even less with virtual humans with dementia. Examples of early work focused on human interactions using IVR are Mel Slater's work on social phobias (Slater et al., 2006) as well as the early works by Professor Nadia Magnenat-Thalmann, where her team succeeded on perfecting facial expression and gestures in virtual humans (Magnenat-Thalmann and Thalmann, 2005). Jeremy Bailenson has also published great work on interactive virtual humans, even going so far as exploring the impact of gender and the ability of virtual humans to persuade the user (Yee and Bailenson, 2007).

However, the application of virtual environments to the area of dementia is still a burgeoning field, primarily focused on creating pleasant virtual worlds for PLWD to escape to, such as a forest (Moyle et al., 2018) or as a means to reduce perceived apathy in people living with cognitive disorders (Ho et al., 2022). Some have directed their virtual interventions to care providers, using virtual environments to help develop empathy in workers by recreating what it might feel like to be living with dementia (Adefila et al., 2016). These significant advances contribute greatly to the field, but there is a need to push the work to interactional and education levels, where trainees learn how to interact with a virtual individual with dementia in an emotionally laden situation. Families and health care providers find it most difficult to manage behaviors when they are actually in the situation that is provoking psychological distress. At the time of the writing of this paper, no IVR tools were identified for addressing staff and families' roles in helping to mitigate the triggers to BPSD.

Unlike other training programs and interventions for BPSD, this study focuses on the development of an IVR tool to train health care providers and care partners as agents for the identification and reduction of social and physical environmental triggers of BPSD. Health education studies using IVR primarily focus on teaching technical skills and rarely do they address emotionally laden interpersonal contexts (Liu et al., 2023). Any effective IVR tool that deals with the stressful interactional aspects of the dementia experience would supply care providers with a platform to practice newly acquired skills in more challenging situations that elicit their emotional reactions. Such IVR sessions would elicit some of the same feelings (e.g., anxiety) that would occur in the real world, thereby better preparing families and health care providers to tactfully address the triggers that are creating anxiety for all. Furthermore, IVR is now a transportable technology which can be shared with many users and even online, making it possible for care partners to participate in the program from home if needed. As with many other IVR interventions, care partners would also be able to practice their skills as often as needed, without the need for a simulated patient to be present, and to overlearn skills if necessary.

A recent report by an Immersive Healthcare Collaborative suggests three principles to consider as scalable, evidence-informed IVR tools are created (Mathew and Mushtaq, 2021). While these may seem evident to the naïve reader, all three principles are rarely adhered to in the development of health-related immersive tools. The first principle suggests that the design and development of the tool be driven by learning needs. That is, learning needs to be met through the IVR tool, as opposed to traditional training approaches to BPSD, need to be clearly defined. For instance, what might a developer want the trainee to learn by interacting with a virtual person with dementia that cannot be met otherwise? The second principle suggests that any tool that is implemented be rigorously evaluated. In the case of dementia, would an IVR training tool for care partners lead to better integration of the learned skills in the real, physical world? How might one collect this evidence? The third principle is that development of any such tool include a collaboration amongst developers and users to foster efficient and effective use of the technology. Applying this principle to the development of an IVR tool for training care partners of PLWD means including input from clinicians, educators and technology developers to develop the best tool possible. A tool that creates a virtual human with dementia could not rely on the knowledge of technology experts without including the input of care partners and experts in dementia.

The overall goal of this project was to examine whether a virtual environment that includes a virtual LTCH resident with dementia in a lifelike situation could be useful and usable for health care providers and care partners to identify potential triggers to BPSD (Study aim #2) while being engaged emotionally with the scene (Study aim #1).

Creating interactive immersive training tools that focus on emotion and interpersonal relationships is complex and time consuming. For this first phase of the study, before a true training tool could be developed where the trainee interacts with his environment, the user was placed in the position of observer. It was important to know if the virtual environment and scenario were convincing enough to users before a more sophisticated, interactive protocol was developed. Professional noticing (Rooney and Boud, 2019) is important in clinical training and is an important first step to clinical learning.

The current project was therefore designed to answer the following research questions: (a) is the virtual environment perceived to be realistic as to engage participants emotionally (Study aim #1); (b) is the virtual environment perceived as easy to use and useful as a potential training tool within the context of BPSD (Study aim #2). Both aims were addressed with descriptive and qualitative analyses. For the descriptive analyses, the Independent Television Commission Sense of Presence Inventory (ITC-SOPI; Lessiter et al., 2001) was used to assess engagement (Study aim #1); the Simulator Sickness Questionnaire (SSQ; Kennedy et al., 1993) and one Yes/No question were used to assess usability and four Yes/No questions were used to assessed usefulness. Qualitative analysis of open-ended questions resulted in a better understanding of participants' perceptions of usefulness and usability of the tool.

## 2 Methods

### 2.1 Participants

A total of 87 participants were included. Twenty-seven were university or community college students in training to obtain a professional designation, 11 were university or community college faculty members teaching courses relevant to gerontology and dementia, 24 were healthcare professionals working with persons living with dementia, and 25 were care partners to persons living with dementia. Students and faculty members were from the University of Ottawa (Faculties of Health Sciences, Social Sciences, and Medicine) and Algonquin College (Personal Support Worker Ontario College Certificate program), both in Ottawa, Canada. Healthcare professionals were recruited from the Perley Health, Elisabeth Bruyère Residence, Saint-Louis Residence, and City of Ottawa Long-Term Care Homes, also all in Ottawa, Canada. Care partners were recruited through presentations at the Alzheimer Society of Ottawa and Renfrew County and Elizabeth Bruyère Memory Disorder Clinic as well as through community family physicians.

To be included, healthcare professionals had to have practical experience with individuals presenting BPSD, students had to have theoretical knowledge of dementia and/or practical experience with BPSD, and university or community college faculty members had to have taught a subject related to dementia. Approval from all relevant and participating Research ethics boards was obtained prior to the study and all participants signed a free and informed consent form.

### 2.2 Procedures

#### 2.2.1 Pre-immersion

Informed consent was obtained and pre-test questionnaires completed (see Section 2.3 for details). Participants were then introduced to the virtual human named Mr. Smith to set the context of the immersion in VR and help establish some attachment with him. A 7-min video showed a (non-virtual) female speaker, posing as Mr. Smith's spouse, providing basic biographical information about him and his granddaughter, his life prior to dementia and the impact dementia had had on their lives. The video is available online as Supplementary material through the following link: Lives lived—Mr. Smith. This fictional biography was based on a collage of real-life experiences shared by various individuals that have accompanied persons with dementia and validated by families and workers at the local Dementia Society. Participants were then briefed on how to use the IVR system, assisted with the fitting of the head mounted display (HMD), and the following verbal instruction was given: “*During your entire experience, please be attentive to the interactions between Mr. Smith and his granddaughter and anything else you might notice going on around you*.” Finally, the IVR scenario was launched.

#### 2.2.2 Immersion—phase one (preparation)

The preparation phase was designed to habituate participants to the VR equipment, familiarize themselves to the immersive environment and, most importantly, experimentally induce physiological arousal and stress. The intention in elevating the degree of physiological arousal and stress was to trigger emotional states that might cloud participants' abilities to remain calm and collected during the second phase of the immersion and replicate some of the real-world stress health providers and care partners might experience prior to interacting with residents with cognitive problems. Working as care partners with individuals with dementia who experience BPSD has indeed been documented as potentially stressful and potentially impacting care delivery (Hazelhof et al., 2016). Note that participants could not see a virtual representation of their body in the virtual environment.

**Figure 1:**
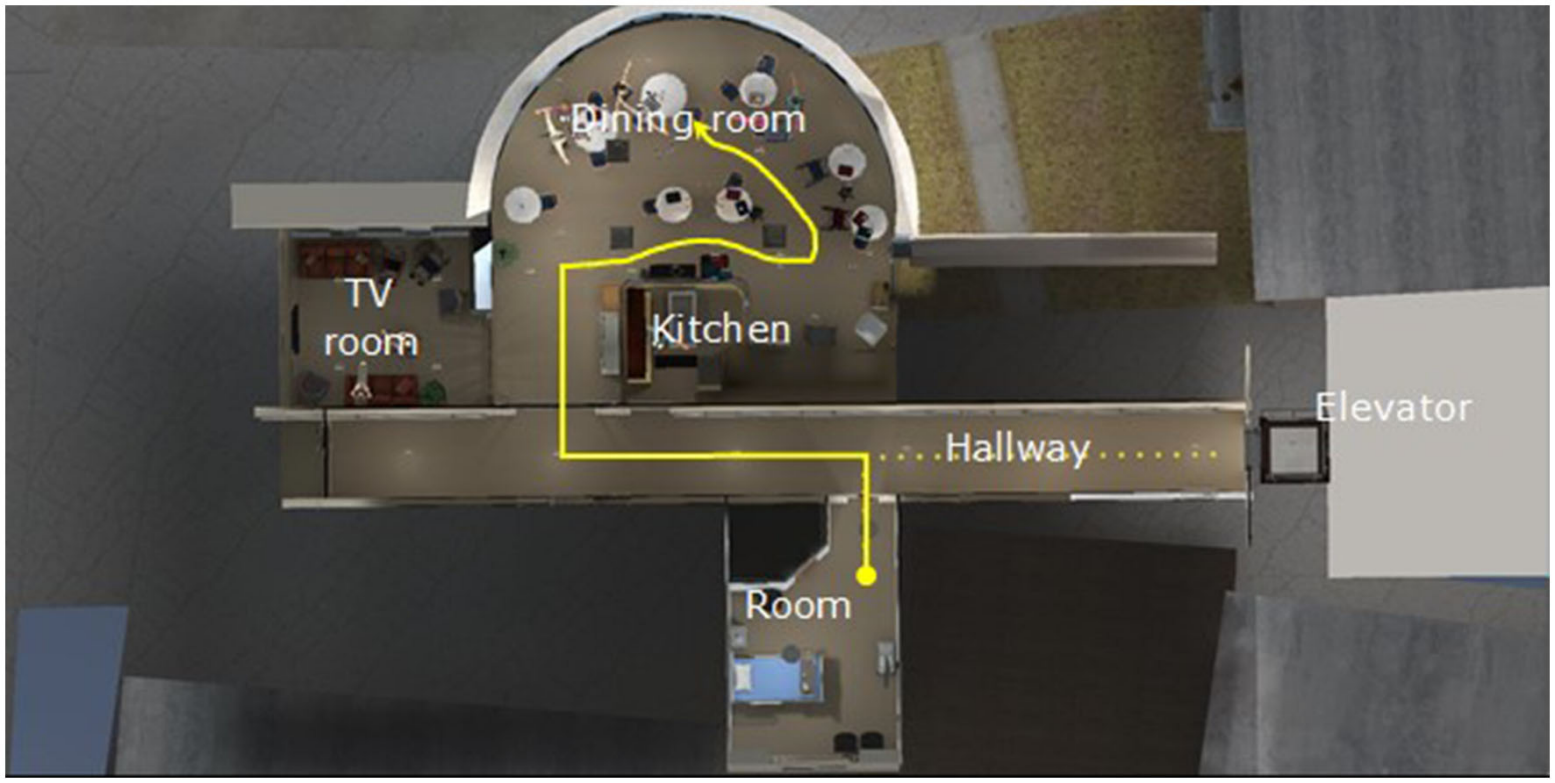
Bird's eye view of the immersive path, starting with the dotted line from the elevator.

The immersion began with participants being in an elevator arriving on the fifth floor of a LTCH (Figure 1) and receiving pre-recorded instructions about how to move and navigate in IVR using the controllers. The participants then exited the elevator and followed a series of dots on the floor to a bedroom. There, participants were given time to accommodate to IVR and to practice moving about in the virtual environment. Once participants indicated being ready to begin, two n-back (Kirchner, 1958) cognitive exercises were administered (one n-1 and one n-2, see Section 2.3.2).

#### 2.2.3 Immersion—phase two (virtual scenario with Mr. Smith and his granddaughter)

Once participants indicated being ready to continue, they followed another series of dots on the floor that led them out of the bedroom, down a hall, into a large dining room of the LTCH, and to a table at which were sitting the grandfather living with dementia (Mr. Smith) and his granddaughter.

**Figure 2:**
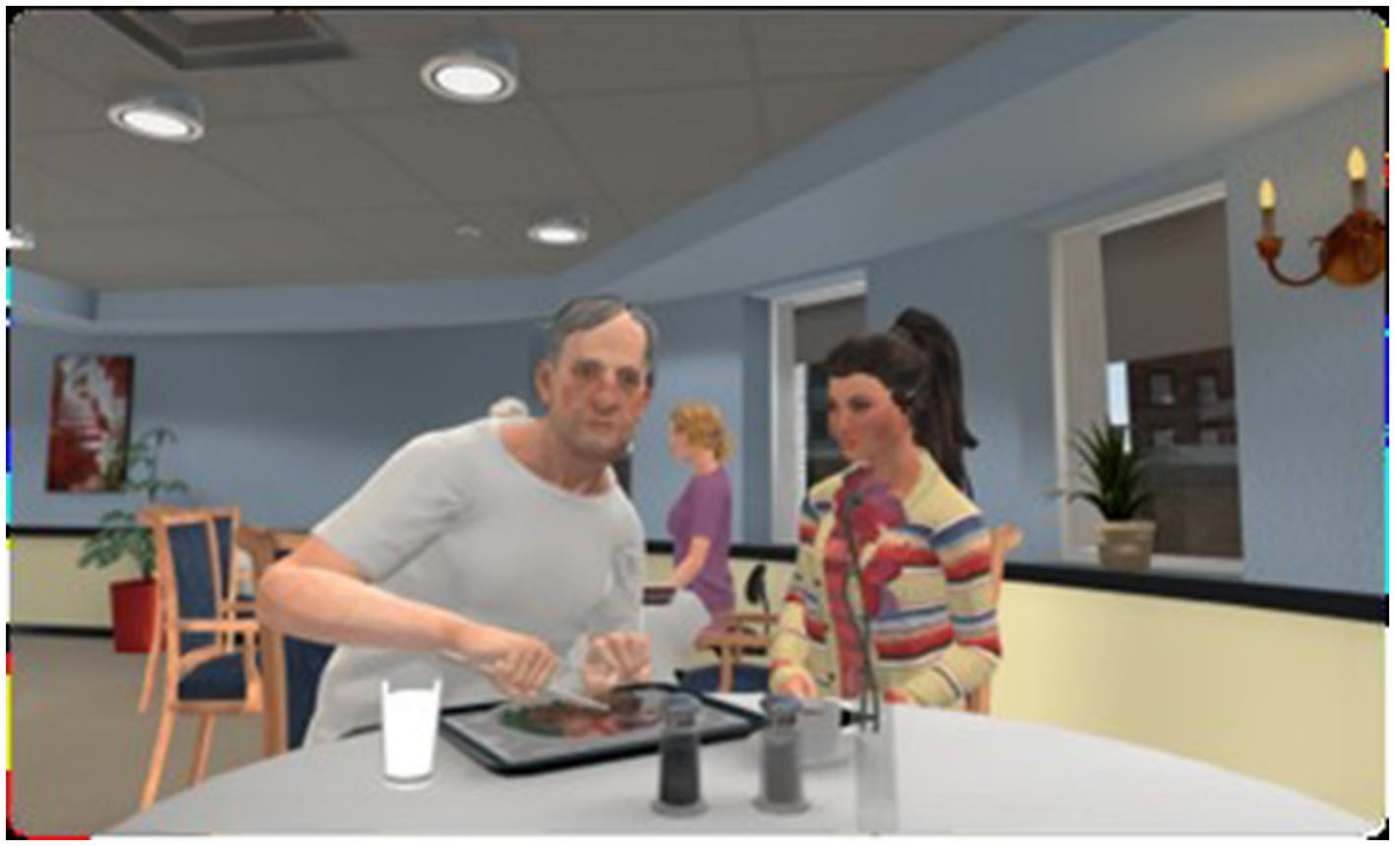
Screenshot of the virtual grandfather and granddaughter at mealtime as seen from the point of view of the user immersed in VR.

**Figure 3:**
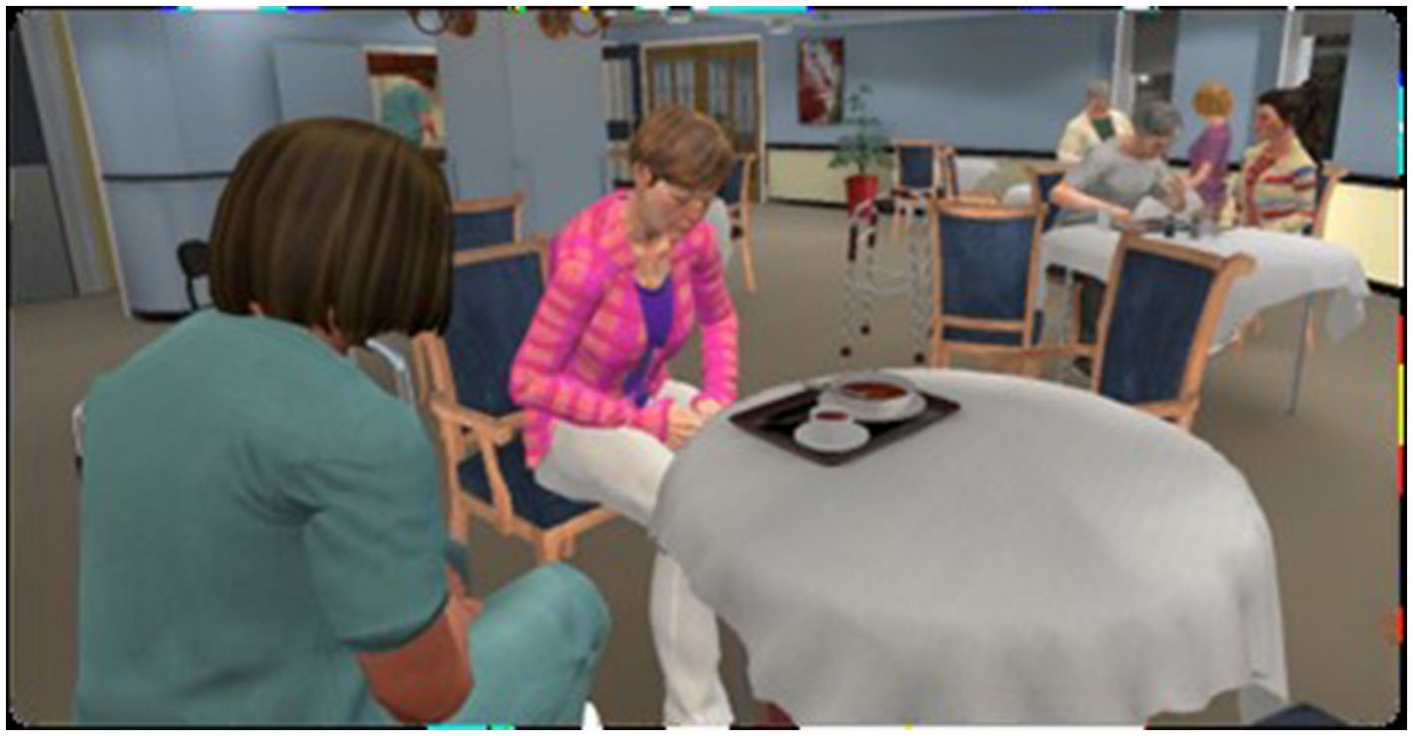
Screenshot of the virtual environment illustrating the presence of additional virtual humans at mealtime.

Once in the dining room, participants were assisted to physically sit down on a chair at the virtual table to observe, as a virtual visitor, the interaction between Mr. Smith and his granddaughter during mealtime. Other virtual residents and health care providers were also present in the virtual environment (see Figures 2, 3). During the 10-min interaction, the virtual dining room was lively with people interacting at other tables (conversations, sometimes screaming from other residents and requests “to go home”), public announcements on speakers, television playing loudly, clanking and sometimes breaking dishes, other virtual humans in the TV room and a clerk working in the kitchen. Mealtimes have been documented to be unpleasant for PLWD and have been the focus of several interventions (Heikkilä et al., 2022).

Figures 2, 3 illustrate the main virtual characters in the scenario. Mr. Smith is a resident living with dementia. The scenario unfolds as he becomes increasingly frustrated with cutting his food and with other environmental cues and triggers such as loud music and TV noises, other residents resisting care, the behavior of his granddaughter and the cold temperature in the room. He expresses his frustration as much through body language as through spoken language. These reactions escalate until he finally lashes out at the participant seated at the table, threatening the participant with the knife he is using to cut his food. The other virtual character interacting with Mr. Smith is his granddaughter. Her behavior is exemplary of how one should not act with a person living with dementia. She speaks too much, speaks too fast, lacks patience and constantly attempts to intervene in a patronizing way, not respecting her grandfather's independence.

Seven key pivot points were created in the scenario in addition to various facial expressions, gestures and words indicating increasing levels of frustration. The key points of escalating tension in the scenario are: (1) arrival of the participant at the table, (2) food on the side of the Mr. Smith's mouth that the granddaughter attempts to clean, (3) the granddaughter's cell phone ringing, (4) water that falls on Mr. Smith's shirt as he tries to drink from a glass, (5) Mr. Smith showing signs of being cold, (6) Mr. Smith being offered help by the granddaughter to cut his meat and the granddaughter's phone ringing a second time, and (7) Mr. Smith beginning to eat with his knife rather than his fork. Each of these pivot points represent critical opportunities for trainees to learn how to detect and defuse situations that could lead to BPSD. The scenario was created with the input of care partners to persons living with dementia, the local Dementia Society and clinicians working in the community with persons living with dementia. They were consulted as the scenario was being developed. The scenario is available online in three parts as Supplementary material through the following links: Part 1 VR protocol, Part 2 VR Protocol, Part 3 VR Protocol.

#### 2.2.4 Post-immersion

Once the scenario was complete, the researcher assisted participants in taking off the HMD and ensured that participants' sense of balance was stable. Participants completed three questionnaires and a series of qualitative open-ended questions on the potential use of this tool to identify triggers to BPSD, usability of the tool and the potential of using this tool to train health care providers and care partners. Finally, participants were given the opportunity to provide verbal feedback about the experience and ask questions about the project.

### 2.3 Materials

#### 2.3.1 The IVR system

The IVR system was set-up in a room with an immersion space of about 220 cm by 190 cm. The virtual environment was displayed by an HTC VIVE system running on an Intel Core i7-6700 K processor (4-Cores, 8 MB Cache, Turbo Boost 2.0, Overclocked up to 4.2 GHz), an NVIDIA GeForce GTX 980 with 4 GB GDDR5 graphic card, 256 GB M.2 PCIe SSD (boot) and 2TB 7200 RPM SATA 6 Gb/s (storage) hard drives, with memory of 16 GB DDR4 at 2,133 MHz, and a Windows 10 Home 64 bit operating system. Images were projected in stereoscopy in the HTC VIVE HMD and spatialized sound was delivered to participants in Cyber Acoustics stereoscopic headphones. The six-degree-of-freedom motion tracking was provided by two VIVE's base stations. Walking forward and backward in the virtual environment was accomplished with the wireless VIVE controllers. Smaller positional adjustments (such as rotations and shuffles) were made by participants' physically adjusting they body position as desired.

#### 2.3.2 The n-back continuous performance task

As introduced in Section 2.2.2, two n-back tasks (Kirchner, 1958) were administered to participants at the end of the first phase of the immersion to induce physiological arousal and stress. The n-back is a continuous performance task where participants are presented a sequence of audio stimuli one-by-one. For each stimulus, participants must quickly decide if the current stimulus is the same as the one presented n turns ago. The exercise, performed while immersed in VR, lasted ~120 s. It began with general instructions for the exercise, followed by a 1-back task, then a 2-back task.

#### 2.3.3 Descriptive information

An 11-item questionnaire collected basic demographic data (e.g., age, sex, and education) and documented the participants' levels of experience with dementia and IVR.

#### 2.3.4 Immersive tendencies questionnaire (ITQ)

The ITQ assesses trait-like characteristics that predispose people to feel present in IVR (Witmer and Singer, 1998). It consists of 18 items rated on a seven-point scale (0 = never; 6 = often) providing a total score and four subscale scores (Cronbach's alpha = 0.78): Focus (the ability to concentrate and to ignore distractions; five items), Involvement (the feeling of being caught up by stories and movies; five items), Emotion (the intensity of the emotions evoked by stimuli such as movies; four items), and Play (the frequency of playing video games; three items). Although the ITQ was developed decades ago, it remains a useful measure of how much people are receptive to immersive experiences (Weibel et al., 2010). It was administered pre-immersion to characterize the sample.

#### 2.3.5 Independent television commission sense of presence inventory (ITC-SOPI)

The ITC-SOPI measures thoughts and feelings experienced while in the immersion so as to document presence (Lessiter et al., 2001). It is composed of 44 items, scored on a scale from 1 to 5 (1 = strongly disagree, 5 = strongly agree), addressing: (a) Spatial Presence (sense of physical placement in the mediated environment, interaction, and control over different parts of the environment; 19 items); (b) Engagement (tendency to feel psychologically involved and to enjoy the content; 13 items); (c) Ecological Validity/Naturalness (tendency to perceive the mediated environment as lifelike or real; five items); and (d) Negative Effects (tendency to have adverse physiological reactions; six items). The authors of the inventory reported internal consistency coefficients (Cronbach's alpha) ranging from 0.94 (Spatial Presence) to 0.76 (Naturalness). The ITC-SOPI is the second most-often used questionnaire measuring the subjective feeling of presence (Souza et al., 2021) and was administered post-immersion.

#### 2.3.6 Simulator Sickness Questionnaire (SSQ)

The SSQ measures the level of unwanted negative side effects induced by immersions in VR (commonly referred to as “cybersickness”) felt by participants (Kennedy et al., 1993). It consists of 16 items and measures, via a four-point scale (0 = not at all, 3 = severely), the intensity of various negative side effects (e.g., nausea, eye strain; Cronbach's alpha = 0.86). The SSQ is the most often used measure of IVR side effects and was scored according to the revised procedures recommended by Bouchard et al. (2021) for the Nausea, Oculomotor and Total scores. It was administered pre- and post-immersion to document usability.

#### 2.3.7 Perceived usability and usefulness

Perceived usability and usefulness were documented with the following questions answered with dichotomous Yes/No choices, with one question about usability and three about usefulness:

- *Was the virtual reality module easy for you to use?*
- *Do you think this type of virtual module was useful in helping you learn how to respond to the behaviors of people with dementia?*
- *Would you like to see virtual reality used in training for responsive behaviors in the curriculum for future healthcare providers?*
- *Would you like to see virtual reality used in training for responsive behaviors for people working in long-term care?*
- *Would you like to see virtual reality used in training for responsive behaviors for people living with people with dementia in the community?*

#### 2.3.8 Qualitative assessment

Perceived usability and usefulness were further explored with 13 open-ended pencil-and-paper questions addressing usability (1–2), usefulness in eliciting environment triggers (3–5) and usefulness and usability for training (6–13):

1. *What are your thoughts and impressions before trying VR out? Other comments?*
2. *What are your thoughts and impressions now that you've tried out the virtual reality module?*
3. *What do you think the granddaughter in this scenario would have or should have done differently?*
4. *What would you or could you have done in this situation?*
5. *Are you able to identify possible triggers that may have led to the virtual person with dementia acting the way he did? What do you think these could be?*
6. *Do you think this type of virtual module was useful in helping you learn how to respond to the behaviors of people with dementia—please explain your answer*.
7. *Would you like to see virtual reality used in training for responsive behaviors in the curriculum for future healthcare providers—please explain your answer*.
8. *Would you like to see virtual reality used in training for responsive behaviors for people working in long-term care—please explain your answer*.
9. *Would you like to see virtual reality used in training for responsive behaviors for people living with people with dementia in the community—please explain your answer*.
10. *Was the virtual reality module easy for you to use—please explain your answer*.
11. *What appealed to you the most about the virtual reality module?*
12. *What did you like least about the virtual reality module?*
13. *What challenges did you encounter while using technology itself?*

### 2.4 Data analysis

#### 2.4.1 Quantitative data

Descriptive statistics were conducted on age, gender, years of education, immersive tendencies, presence, simulator sickness and the five Yes/No questions. Correlations were performed to explore the relationship between subscales of the ITQ, ITC-SOPI, and post-immersion SSQ. Paired-samples *t*-tests were conducted to examine differences in pre- and post-immersion for the Nausea, Oculomotor and Total SSQ scores.

#### 2.4.2 Qualitative data

Participants' responses to the open-ended questions were coded as a function of their perceptions of the usability of the tool, its usefulness with regards to environmental triggers and its usefulness and usability for training. A codebook was created with definitions for each of the codes. Twenty-five percent of the responses were then recoded by a second researcher and frequencies for each code were tabulated. No quantitative inter-rater agreement score was obtained but any disagreements were discussed until there was consensus. The results were triangulated with the results of the Yes/No questions, the ITC-SOPI (for engagement) and the SSQ (for usability) to document credibility of the qualitative results.

## 3 Results

Eighty-five participants tested the application. The participants ranged between 24 and 82 years of age with a mean age of 46.55 (SD = 16.91). There were 11 (12.9%) participants who identified as male and 74 (87.1%) as female. On average, they completed 18 (SD = 3.9) years of education. Twenty-three were health care providers (personal support workers and nurses) working with people living with dementia, 23 were family or friend care partners to someone living with dementia, 10 were professors and 29 were students in the health, medical and social sciences or in training to become personal support workers. Sixty participants (70.6%) had had some training in aging, 58 (68.2%) in dementia specifically, 77 (90.6%) had experience interacting with persons living with dementia, 35 (41.2%) had some training on BPSD and 24 (28%) of the same had ever used IVR before. Participants had an average total score of 51.78 (SD = 14.52) on the ITQ; and 19.71 (SD = 3.95) on the ITQ focus, 13.13 (SD = 6.04) on the ITQ involvement, 11.49 (SD = 5.11) on the ITQ emotion, and 3.56 (SD = 3.76) on the ITQ play subscales.

### 3.1 Quantitative results

Engagement was documented with the ITC-SOPI measure of presence. Participants reported an average score of 4.20 (SD = 5.67) on the Spatial Presence subscale, 4.54 (SD = 8.34) on the Engagement subscale and 3.78 (SD = 0.57) on the Ecological Validity and Naturalness subscale. The average score for the Negative Effects subscale was 2.29 (SD = 0.68).

Paired-samples *t*-tests revealed significant increases in unwanted side effects induced by the immersion in VR for the Nausea subscale [*t*_(81)_ = 3.98, *p* < 0.001, Cohen's *d* = 0.44], the Oculomotor subscale [*t*_(80)_ = 2.37, *p* = 0.02, Cohen's *d* = 0.26], and the Total score [*t*_(79)_ = 3.64, *p* < 0.001, Cohen's *d* = 0.41]. See Table 1 for the raw scores for these subscales and Table 2 for correlations between the SSQ at post-immersion and the other scales and subscales.

**Table 1:** Descriptive statistics for results on the Simulator Sickness Questionnaire (SSQ)—raw scores.

	**Pre-immersion**		**Post-immersion**	
	**Mean**	**Std. dev**	**Mean**	**Std. dev**
SSQ raw scores—Nausea subscale	0.51	1.27	1.36	2.27
SSQ raw scores—Oculomotor subscale	1.46	1.62	1.99	2.28
SSQ raw scores—Total sore	1.98	2.47	3.27	4.10

**Table 2:** Correlations between immersive tendencies, presence and unwanted negative side effects induced by the immersion in virtual reality.

**Variable**	**1**	**2**	**3**	**4**	**5**	**6**	**7**	**8**	**9**	**10**	**11**	**12**
1. ITQ Total	-											
2. ITQ Focus	0.72^**^	-										
3. ITQ Involvement	0.85^**^	0.51^**^	-									
4. ITQ Emotion	0.77^**^	0.38^**^	0.54^**^	-								
5. ITQ Play	0.64^**^	0.35^**^	0.40^**^	0.35^**^	-							
6. SOPI Spatial	0.18	0.39^**^	0.073	0.02	0.07	-						
7. SOPI Engagement	0.24	0.34^**^	0.21	0.11	0.11	0.58^**^	-					
8. SOPI Ecological	0.13	0.32^**^	−0.005	−0.03	0.16	0.70^**^	0.52^**^	-				
9. SOPI Negative	0.02	−0.17	0.01	0.13	0.09	−0.15	−0.25^*^	−0.16	-			
10. SSQ Nausea	0.25^*^	0.17	0.26^*^	0.18	0.18	−0.06	−0.14	−0.17	0.50^**^	-		
11. SSQ Oculomotor	0.16	0.08	0.14	0.20	0.08	−0.19	−0.11	−0.30^**^	0.47^**^	0.71^**^	-	
12. SSQ Total	0.23^*^	0.12	0.22^*^	0.77^**^	0.64^**^	−0.16	−0.15	−0.28^*^	0.51^**^	0.92^**^	0.93^**^	-

ITQ, Immersive Tendencies Questionnaire; ITC—SOPI, Sense of Presence Inventory; SSQ, Simulator Sickness Questionnaire—raw scores.

^*^p < 0.05.

^**^p < 0.01.

Based on the Yes/No questions, results showed that 74 (87.1%) participants reported the VR module easy to use. Sixty-one (71.8%) participants reported that the virtual module was useful in helping them learn how to respond to the behaviors of PLWD. Seventy-five (88.2%) participants reported they would like to see virtual reality used in training for BPSD in the curriculum for future health care providers. Seventy-two (84.7%) participants reported they would like to see virtual reality used in training for responsive behaviors for people working in LTCH. Sixty-eight (80%) participants reported they would like to see virtual reality used in training for BPSD for PLWD in the community.

### 3.2 Qualitative results

The **ease of use** of the IVR training module was reported as good despite some participants never having used this type of technology before. By far the most common code was “*easy to use*.” Comments reflected that the tool was easy, simple, intuitive and straightforward to use, with clear instructions.

- *Yes. Really great experience. I have wondered what it would be like as I have seen glimpses of people on TV wearing similar equipment. Clear instructions*.
- *Yes, the controls were straightforward*.
- *It is reasonably simple. I don't play computer games so I don't bring many skills to the project*.
- *Control key, instructions were clear. Easy*.
- *Even though I don't normally use VR, it was fairly easy*.
- *It was easier than I had anticipated* + *would become even more so after a few sessions*.

A small number of participants did, however, report that the VR helmet was somewhat bulky and sometimes uncomfortable to wear. The most common negative comment about usability was related to moving around in the VE. It took a little time for some participants to get used to the sensory conflict between what they saw and how their bodies were reacting. Participants did experience motion sickness, disorientation, dizziness and balance issues as expressed in their words.

- *I got used to it—but not 100% as I could not master the speed of walking*.
- *It was a bit dizzying at first…*
- *I felt like all of my senses were being bombarded at once. I was disoriented as to where my body was in the lab room*.

Participants' overall reactions to usefulness of the tool to **engage** them in the VE were positive. They reported that the multiple sensory inputs made the VE highly immersive, and the non-human-like elements of the VE (rooms, objects, etc.) were perceived as realistic. During immersion, the participants felt present in the virtual LTCH home, thus forgetting that, physically, they were in a research laboratory. The interactions between the virtual persons were described as real-life-like, sometimes eliciting feelings of “déjà-vu.” Many participants reported becoming emotionally involved when witnessing the interaction between the granddaughter and the PLWD (e.g., frustration toward the granddaughter and empathy for the PLWD).

- *I was emotionally involved in the interaction between Mr. Smith and his granddaughter and felt threatened by the pointed knife*.
- *VR is very lifelike and felt realistic to the point where I wanted to get involved in the scenario*.
- *So, I practiced as a clinical social worker for many years and I felt my body assuming that position. I wanted to interact and speak—respond to the situation that was unfolding*.
- *I thought it was very impressive in that I did feel “part” of the scenario. I found myself speaking out loud as if I could actually influence the “characters” in the scene. I found that Mr. Smith* + *his granddaughter actually felt quite real*.

However, some participants reported limitations regarding the VE. For example, it was reported that the movement and physical characteristics of the virtual persons lacked realism, and a small number of participants described them as too “cartoon-like.”

A large majority of participants reported that this type of IVR application could be a **useful training tool** to help participants better address BPSD and their triggers. More specifically, participants reported that IVR could be helpful in bridging the gap between theory and practice by exposing the participant to a safe environment and to the sequence of typical reactions (verbal and body language) that are precursors to BPSD. However, many participants deplored the fact that the application put the participant solely in the role of an observer, and that they were not able to interact with the virtual characters.

- *This gives a visual, life-like representation of what it is actually like to deal with people with dementia. It allows for practice of different situations and gives time to think of how to interact or prepare yourself in a safe environment*.

The most-mentioned positive point was related to the fact that the participant actually felt like they were living the situation. Due to the sense of **feeling present** in the virtual LTCH home and the feeling of co-presence with the virtual persons, the participants communicated that they were emotionally involved, more-so than when watching a training video, and similarly to what would happen in a real-world situation. Participants used terms such as “*rich immersive experience*,” “*exposed to what it is really like*,” “*excellent way to feel engaged with the situation*,” and “*nothing compares to experiencing something*.”

- *During my undergrad there was a lot of opportunity to practice practical, clinical skills (ex: IV insertions, blood product administration, etc.,) and we were then tested on our ability to perform the skill in accordance with agency policy* + *procedure but there was no lab to simulate how to interact with someone with dementia/no labs on how to have a therapeutic interaction with patients*.
- *All family physicians, nurses, PSW, recreation, physiotherapy students as well as staff should have VR training for better preparation and understanding*.
- *I believe that it should be part of training … Reading or being told what to do is different from seeing it in a life-like experience*.
- *This could be part of their yearly performance appraisal. Continuing education*.

For instance, part of our inquiry was to see if participants could detect from our scenario **what actions might not be desirable in the interaction** and what they, as participants, would have done differently had they been able to interact with the virtual characters. To the question “*What do you think the granddaughter in this scenario would have or should have done differently?”* the follow responses were noted:

- *She did not understand the full situation. Her grandfather is very autonomous (likes to do things on his own) and she kept insisting on helping. He didn't want her help*.
- *Turn off the phone. Allow grandpa more independence* + *dignity. Engage grandpa by asking open-ended questions. Speak kinder, show more compassion*.
- *She should have listened to what her grandfather was saying* + *kept in mind that he had been his own person, making his own decisions all his life so that it would be difficult for him to concede his independence to others*.
- *Mr. Smith seemed to be affected by all the simultaneous noises and information that were co-occurring at the scene… I would have tried to keep my non-verbal signs away from feelings of embarrassment, frustration, disappointment, boredom*.

When asked, “*What would you or could you have done in this situation?”* respondents said:

- *I would have offered the grandfather a napkin in a way where you are giving him a choice rather than being demanding*.
- *I could introduce myself and explain why I am there and offer options to the patient. Would you like to cut meat yourself or do you want me to do it?*
- *I would have turned off background music. Offered clothing protector. Let the resident do as much as he can for himself to promote a sense of independence (always focus on what the resident can do—not on his/her deficits)*.

It was evident from these participants' observations that the scenario was sufficiently well-designed to elicit convincing responses that identified triggers to behavior and potentially appropriate intervention approaches.

## 4 Discussion

Overall, the results of this precursory project support the use of an IVR application as a training tool for those who interact with people living with dementia. In the perceptions of the participants, the VE was realistic enough to engage them (Study aim #1), the tool was easy to use (usable) and useful as a future training tool within the context of the identification of triggers of BPSD under potentially stressful conditions (Study aim #2).

The scenario was found to be believable and engaging to the participants. Scores on the measures of presence are comparable to other simulations in VR (Baus et al., 2019, 2022; Yildirim et al., 2019; Simon et al., 2020; Bouchard et al., 2021) and supported by the qualitative descriptions of many participants who wanted to intervene during the observations of the virtual interactions. Not only does this signify its potential use for training on strategies for identifying the triggers to BPSD but the fact that participants were engaged and highly present suggests that it might lead to changed interactions and interpersonal approaches with PLWD, as health care providers and care partners learn how their own actions can be triggers to BPSD.

The recent COVID-19 pandemic has made the need to transform care approaches in LTCH one of Canada's top priorities. Early in the pandemic, Canada was seeing over 80% of related deaths occurring in these settings (Akhtar-Danesh et al., 2022). While its rates were among the highest worldwide, many countries were unprepared, laying bare the need to adhere to decades of reports promoting a change in attitude and approach to the kind of care given in these collective dwellings increasingly filled with residents living with dementia. The engagement of the participants in the current project suggests that virtual scenarios can be created to elicit more desirable interactions that reflect a more resident-centric approach. Minimally, it can be used to help health care providers and care partners see themselves as environmental agents themselves, along with elements of the physical environment as capable of eliciting or diminishing BPSD that are responsive to these environments.

While the majority of participants found the tool easy to use and the instructions clear, as reflected in their responses to open-ended questions, some reported feeling uneasy or dizzy in the VE. These qualitative findings triangulate with the statistically significant increases in side effects after the immersion, as measured with the SSQ. These results were not surprising given that the mean values post-immersion are within the expected range (actually, below average) for general immersions in VR (Bouchard et al., 2021), lower than immersions conducted during psychotherapy for anxiety disorders (Bouchard et al., 2009), and lower than scores reported when people complete the SSQ after a stressor induced *without* any immersion in VR (Bouchard et al., 2021, Study 2). SSQ scores significantly correlated with participants' tendencies to experience strong emotions and play videogames, as measured with the ITQ. These correlations and the low intensity of SSQ symptoms suggest the effects observed on the SSQ may be related to emotions induced by the stressful nature of the interactions with the virtual humans and the participants. Such a finding is consistent with results showing that stress and anxiety influence SSQ scores (Bouchard et al., 2009, 2021) and warrants more attention in future studies. It might also indicate that the VE environment that was created for this study closely mimics the realities of care for some health providers and family members.

A large proportion of participants agreed that it could be used for training purposes but in order to be an appropriate training tool, attention will need to be brought to creating adjustable levels of difficulty, including the possibility of repeating scenarios for learning purposes and including a mechanism for evaluation. Not only would such a tool empower health care providers to have a learning mechanism by which they can move forward at their own pace, but it could also serve as an assessment tool for soft skills. Identifying the triggers to BPSD and learning how to respond in stressful situations would go a long way in improving quality of life for both PLWD and their care partners. It reinforces and demonstrates the role played by both social and physical triggers to the manifestation of BPSD in persons living with dementia.

### 4.1 Limitations and future considerations

The current results suggest that there is merit in creating immersive environments that use virtual humans such as Mr. Smith, the granddaughter and other cast members to create realistic scenarios that engage, are appropriate and useful for training health care providers and care partners in the management of BPSD. The results do not confirm whether training using these means is superior to other training methods, nor does it yet offer a gradation of levels of complexity for training, an interactive function, and a feedback mechanism. The study does, however, offer the groundwork for building these next steps. Moving forward, the learner goals for each of the categories of participants (i.e., formal staff, family care partners, professors and students) will need to be identified. Interested parties could build on their existing training programs and incorporate them into a virtual format. Locally, the Champlain Dementia Network and Dementia Society of Ottawa and Renfrew County has prepared an online *Dementia Companion Certificate* which identifies clear learner goals. Such goals, for example, could be used in the next iteration of a virtual tool.

Some preliminary lessons were learned regarding the adjustments to be made for future applications. From the technical standpoint, the unwanted negative side effects induced by the immersion in VR experienced by some participants suggests that for future applications, it may be appropriate to design a scenario that allows participants to remain seated during the entire immersion. This adjustment to the protocol would reduce the sensory conflict that results from moving about in the VE.

The observations from participants that the scenario was not interactive enough was not a surprise to the research team. It was the planned second step in the development of the software. However, given the “pilot nature” of this project, putting the participant in the role of an observer was the logical first step to gather feedback. The advent of generative artificial intelligence programs facilitates the development of complex interaction with and among virtual characters. Moving forward, however, additional challenges will need to be faced in addition to making the tool more interactive.

The results do suggest that IVR may indeed be a viable alternative to in-person high-cost training with simulated patients. Indeed, one concern about the feasibility of using an IVR module for training regarded the cost, especially if it was to become more interactive. While it would imply a significant upfront cost, once developed, it could be widely distributed at relatively low cost. The benefits and advantages, should it be successful, would far outweigh the costs. The study was conducted with a setup that was non-mobile. To cost out any future systems would necessitate building an interactive element to the scenarios, allowing users to try different strategies and obtaining evaluations of their attempts. At the time of the writing of this article, a cloud platform might be the best solution so as to allow access to users both in homes and in the community. Such elements would need to be added before the tool is implemented into homes.

In short, participants felt engaged and saw the potential of the tool. Future steps would include making the tool more interactive, creating more scenarios with various BPSD, rendering the tool more accessible and scaling the tool to include precise learning objectives and evaluation methods. A fully developed, interactive tool could offer a superb steppingstone between learning in a classroom and practicing real world human interactions as they relate to the identification of environmental triggers as precursors to BPSD and strategies for diminishing their impact.

## 5 Conclusion

It is now recognized that many of the behavioral symptoms of dementia are the result of unmet needs that are responsive to environmental challenges. It therefore behooves those who live and work with individuals experiencing dementia to understand what those environmental triggers might be for any given individual and to learn strategies for reducing their impact. Several successful programs exist that help families and staff do just that. However, anecdotal evidence from both family care partners and staff suggests that these strategies may be difficult to remember in situations of stress and anxiety. As a result, faced with the demands of real-life situations, care partners and staff may be unable to put into practice skills they learned in less interactive classroom settings. The current study addresses this issue, motivated by the fact that immersive virtual reality (IVR) has proven to be effective in creating training tools that require strategies during stress and anxiety-provoking situations. While IVR has been used in the area of dementia to either create pleasant environments for people living with dementia or to simulate what it might be like to have dementia, there is no tool using IVR that trains care partners and staff to identify triggers to the behavioral symptoms while applying appropriate strategies. The current study is a solid first step in creating such a tool. The participants were found to be engaged as measured through an immersive tendencies instrument and through qualitative questions. Participants could identify triggers to the behaviors and judged the tool as having potential to be used for training in stressful situations. The tool and instructions were found to be easy to use and the levels of simulation sickness were comparable to other studies. These results offer evidence that a believable tool can be created that simulates real-life situations where care partners and staff practice learned strategies for interacting with people living with dementia. The study supports the idea that future tools can be created where environmental demands are incrementally augmented so that those who interact with persons living with dementia will be able to respond to unmet needs notwithstanding the stressful demands placed on them.

## Data availability statement

The data for this study are available upon request addressed directly to the Research Ethics Boards of the lead institution (ethics@uottawa.ca) which must first approve the request. If the request is approved, anonymized data supporting the conclusions of this manuscript will be made available by the corresponding author.

## Ethics statement

The studies involving humans were approved by University of Ottawa Research Ethics Board. The studies were conducted in accordance with the local legislation and institutional requirements. The participants provided their written informed consent to participate in this study. Written informed consent was obtained from the individual for the publication of the identifiable image in the video included in this article.

## Author contributions

LG, AR, SB, and LM co-designed the study and interpreted the results. M-CR created the virtual human and scenarios in conjunction with SB and with input from LG. LG supervised the data collection. LG, AR, and SB wrote the paper and produced the final draft. All authors contributed to the article and approved the submitted version.
