# Supplementary material for: The usability of virtual reality to train individuals in responding to behaviors related to dementia
Source: Front Dement. 2024 Jan 8;2:1237127. doi: 10.3389/frdem.2023.1237127 (PMC11285664; doi:10.3389/frdem.2023.1237127)
Supplement: Supplementary file 1 [file Data_Sheet_1.pdf]

Scenario

Grandfather and granddaughter

Overview: Granddaughter (GD) interacts poorly with her grandfather, a person living with dementia (PLWD) in a long-term care home. There is an indication that there is an increasing number of cues that the grandfather is becoming progressively more irritated with the granddaughter, with the environment, and his ability to eat.

General behaviour of PLWD

PLWD tends to maintain a hunched over posture. He acts tired, and keeps re-positioning himself in his chair, as if uncomfortable or even in pain. On and off, he may rub his knee with his hand. He is easily distracted and disturbed by environmental events (people passing by, sounds, etc.).

When drinking, PLWD brings glass very slowly and unsteadily to the mouth.

PLWD has trouble choosing the appropriate utensils to eat, and he shows signs of not visually recognizing how to use the knife and fork. When eating, PLWD seems very concentrated on the task. He has difficulties picking up the food on his fork, and cutting meat is difficult for him. These difficulties frustrate him, and he may stop eating altogether and stare at his plate in frustration before carrying on.

PLWD is resistant to help. When help is offered, he tends to show disapproval by:

(1) adopting a facial expression of closure (e.g., frowning); and/or
(2) shaking head; and/or
(3) turning away his head; and/or
(4) shifting his body away; and/or
(5) voicing his disapproval; and/or
(6) threatening; and/or
(7) physically attacking.

This does not occur all in one scene but the grandfather shows signs of progressively cascading into these behaviours.

General behaviour of GD

GD is well intentioned but acts inappropriately. She wants to help but in doing so, she tends to get into PLWD's bubble (e.g., by reaching in and touching). By helping she is making it more evident that the PLWD cannot do this on his own – which only frustrates him more. The constant talking – looking at phone – becomes irritating – has trouble with silence so fills in gap with speech.

SCENE – PRE-ARRIVAL AND ARRIVAL OF VISITOR

Visitor walks past kitchen.

Sound of dishes crashing down and breaking in the kitchen.

As visitor walks past tables, conversations between nurses and other PLWDs can be overheard; relatively subdued crying and yelling can be heard (Note: needs to be loud enough to be annoying but not overly so).

At the table with PLWD and granddaughter, PLWD is eating (as best as he can manage), glances over at GD from time to time.

GD looks on, concerned, and always talking.

PLWD looks in the direction of the loud conversation between the health staff and other PLWDs.

Visitor arrives at table, and sits down (noise continues in the background — "cafeteria noise"; not yelling)

PLWD is a bit startled (not aggressive) by visitor's arrival because he didn't see the person coming.

PLWD gives the visitor the "Who are you and what do you want" look.

PLWD returns to eating, as best he can.

GD - (to visitor) "*Hi, nice to see you again. Haven't been here for a while. He seems to be doing a lot worse than last time I was here.*"

SCENE – FOOD ON SIDE OF MOUTH

There is background TV noise while scene two is going on.

PLWD is eating (as best as he can manage), glances over at GD and visitor from time to time.

GD – "*Grandpa you have a bit of food on the side of your mouth, here let me help you.*"

GD starts to reach for his mouth.

PLWD pulls his head back, looks towards GD and shakes his head.

GD takes hand away (looks at visitor).

PLWD gives her a look of disapproval (frowning) and goes back to eating.

PLWD – "*I am not a baby.*"

PLWD wipes face with hand.

SCENE – CELL PHONE #1

PLWD is eating (as best as he can manage), glances over at GD and visitor from time to time.

Cell phone rings loudly with obnoxious ringtone.

PLWD jumps with fright, then gives the phone the evil look.

GD reaches over to silence phone, texts and then silences the phone.

GD – "*I'm sorry Grandpa. It was my friend.*"

GD looks at visitor smiling (like the text was a pleasant conversation).

PLWD goes back to eating while GD looks on concerned.

SCENE – WATER DRIBBLING ON SHIRT

PLWD is eating (as best as he can manage), glances over at GD and visitor from time to time.

PLWD slowly reaches for the glass, and brings it slowly to his mouth, with difficulty.

PLWD drinks slowly, shaking glass somewhat. His face shows a level of frustration with not being able to drink like he would like.

GD – "*Grandpa, you have water dribbling down on your shirt, let me dry it off for you.*"

GD starts to reach for napkin. (Loud commercial comes in on TV in background)

PLWD turns away from GD.

PLWD – "***NO!***"

GD pulls her arm back and sighs out of discouragement.

PLWD slowly puts glass back on table and begins eating again.

SCENE – GETTING COLD

PLWD is eating (as best as he can manage), glances over at GD and visitor from time to time.

PLWD stops cutting and sits there looking at his food.

PLWD is visibly cold and starts rubbing forearms.

GD – “*Grandpa, are you cold or do you need some help? What can I do?*”

PLWD shakes head.

GD – “*I can go get your sweater.*”

PLWD – “*I am not cold.*”

PLWD picks up utensils and starts eating again.

(Normal “cafeteria” noise.)

SCENE – HELP WITH CUTTING MEAT

PLWD is eating (as best as he can manage), glances over at GD and visitor from time to time.

PLWD is having increasing difficulty with cutting up meat.

PLWD is very concentrated on this act, and he is visibly getting frustrated with inability to cut the meat and by looking around – he is getting frustrated at the accompanying ambient noise. In other words, it is not just the food that is frustrating him. This means his verbal and non-verbal reactions are increasing in intensity.

GD is watching PLWD as he is struggling and wants to help.

GD – “*Grandpa let me help you with that, let me cut your meat for you.*” (She is getting frustrated that her grandfather is not accepting help)

GD starts to reach for his utensils.

PLWD shifts utensils away from GD and turns head away.

PLWD – “*No!*”

GD pulls her arms back.

GD – “*Grandpa, why won’t you let me help you?*” (frustration)

PLWD pounds on table.

PLWD – “*I can do this!*”

GD – “*Let me help you with that, let me cut your meat for you.*”

(In a distance, we hear someone calling out to get help)

PLWD pounds on table again.

PLWD – *"No!"*

PLWD goes back to eating but avoids looking at GD.

Facial expression shows frustration.

PLWD Lets out a deep sigh, puts down his utensils as if trying to take a break.

GD looks on concerned and looks at visitor.

PLWD picks utensils up again and resumes eating.

Cell phone rings (again) with loud obnoxious ringtone.

PLWD jumps with fright, gives the phone the evil look.

GD – "*I was sure I turned it off. Oh, well."*

GD reaches over to turn off phone, looks over at visitor, and acts as if this is not such a big thing.

PLWD gives GD the evil look.

PLWD is visibly irritated but goes back to eating.

GD looks at visitor again – like 'whatever'.

SCENE – EATING WITH KNIFE INSTEAD OF FORK

PLWD is eating but puts food on knife instead of fork and carries food to mouth with knife.

GD - *(*surprised*)* "*Grandfather, what are you doing?"*

GD - *(*discouraged*)* "*Why are you putting the food on your knife?"*

(looking at visitor) "*I wish I could do something."*

GD - *(*discouraged*)* "*Why aren't you using your fork? You know better than that*."

GD starts to reach for his utensils.

GD – "*Grandpa let me help you!"* – sounds like she is getting mad (this will frustrate the PLWD).

PLWD turns away from GD.

PLWD – "*Leave me alone!"*

GD – "*Come on, I'm just trying to help. Why are you being like this?"*

PLWD gets even more frustrated (another disturbing sound – e.g. clanging of metal from containers and dishes).

PLWD – “*Leave me alone!”*

Note: shivers or signs of pain need to be evident throughout – not just a one-time thing – like the noise

Once again, GD starts to reach for his utensils.

GD – “*Here, let me do it*.”

PLWD pulls utensils away from GD, looks at GD, poundS on table.

PLWD - (yelling) “*NO!”*

GD persists in trying to take the utensils and looks over at visitor.

GD - *(*patronizing and looking back at PLWD*)* “*I know this isn't easy, but I can help.”*

PLWD keeps utensils away from GD.

PLWD pushes back his chair in frustration to put more distance between himself and GD (we hear the noise from the chair).

Glass falls on floor and shatters.

GD pulls back arms, leans back in chair, and lets out a deep sigh.

GD looks at visitor.

GD - (completely frustrated) “*That's it, I give up. Can you please help me?”*

PLWD looks over at visitor and shakes head, as if to say “don't you dare” and “who are YOU?”

PLWD - *(*looking at visitor*)* “*Leave me alone. Why can't you all leave me alone?”*

PLWD gets up, turns towards visitor and swings knife at visitor, visibly frustrated.

PLWD - (yelling) “*NO! I said NO! Leave me alone!”*
