# Supplementary material for: The usability of virtual reality to train individuals in responding to behaviors related to dementia
Source: Front Dement. 2024 Jan 8;2:1237127. doi: 10.3389/frdem.2023.1237127 (PMC11285664; doi:10.3389/frdem.2023.1237127)
Supplement: Supplementary file 2 [file Data_Sheet_2.pdf]

Lives lived - Mr. Jeremy Smith

Today, you will visit my husband, Jeremy Smith, in the dining room of his long-term care home. When you meet him, you will be joining him and our darling granddaughter, Maddie. Before you go in, I want you to meet the man I loved, and still love, before dementia started to rob him of his abilities.

I am going to read this text because there's so much of it that I want to get across and I'm afraid if I become emotional, I'll forget some of it.

Jeremy grew up in Nobleton, Ontario. During his high school years, he played football and basketball. During the summer breaks, he and his best friend, Mark, worked for the T.H.S. moving company. Jeremy was a physically imposing young man. The combination of his physical strength and genuine care for the physical integrity of the items he moved warranted him the nickname: "The mover with the velvet touch". Jeremy's interest in the moving business was not limited to the operational side, and he was quick to learn the administrative side of running such a moving company. To no one's surprise, freshly graduated from university, Jeremy and Mark started their own moving company. They named it, of course, J&M Movers. After a couple of difficult years during which both of them spent countless hours working, the business became financially viable and flourished. It was during these years that Jeremy and I met. He used to tell people that I was "the only person ever able to move him". Together, we had three children: Elizabeth, and the twins William and James. As a family, we were living the Canadian dream: the house with a white picket fence, the two cars, and the bi-annual family vacations. In time, the kids moved out to go to university, and began their professional and family lives far from Nobleton.

When time came for retirement, Jeremy sold his shares of the business and we moved to Montreal to live in a condominium. That way, we would not have the burden of a house with a garden, and we would be close to William and James. Elizabeth was living in New York at that time, and she visited about twice a year. We had lived a very active lifestyle, and retirement was not going to change that.

Jeremy had always been sociable and generous, so now that he had the time, he volunteered to serve meals twice a week at the local mission, and because he loved a great party, he also served on the condo tower's entertainment committee. In the hallway, in the elevator, or in the parking garage, Jeremy never passed up the opportunity to strike up a conversation. Consequently, he knew just about everyone in the building by name.

For Jeremy's 75th birthday, the other members of the entertainment committee organized a surprise party. Even though I was more of an introvert, I was looking forward to it, for Jeremy's sake. At the party, there was mostly the "old gang", but a few more recent move-ins were also in attendance. I didn't know the latter, but expected that Jeremy did, given his social nature. To my surprise, I noticed that the more recent move-ins actually remembered Jeremy's name while he did not remember theirs. While this was somewhat

odd, I thought nothing of it, at first. However, it was a sign of things to come and from then on. I came to notice a cascade of changes in Jeremy's behavior and abilities.

In the beginning, there was just the inability to remember the names of newer acquaintances. Then, came the decreased interest in activities. Jeremy abandoned volunteering, for no particular reason. A few months later, without advising anyone, he stopped going to the meetings of the entertainment committee. When friends of the committee came to visit, he acted emotionally distant. After a while, despite my encouragement to attend, he lost interest in participating in social events altogether. One day, he left to get some milk, and came back two hours later - without the milk. Now I was extremely concerned, but Jeremy resisted the idea of going to see the doctor.
In an attempt to please him, I invited his life-long friend Mark for a visit. It turned out to be a catastrophe: while Mark updated him on all that had happened since their last meeting two years ago, Jeremy mostly stared out the window only responding here and there. When Mark left, Jeremy barely said goodbye to his closest friend. It was then that I decided that consulting a physician was long overdue.

That was two years ago. Today, Jeremy lives in a long-term care home. He is a shadow of the man he used to be. He spends much of his time staring at the TV…, not watching TV, just staring at it. Emotionally, he is almost completely absent. His emotional distance and outbursts of frustration have driven all but the closest of friends and family away. More often than not, those who visit him aren't sure how to act, or whether they are even connecting with him at all.

Once a man who lived life to its fullest, Jeremy Smith is by no means the person he used to be. Looking at him today, one can hardly imagine that he once roamed the football field, running over defenders. One can hardly imagine him working 15-hour days alongside his friend Mark to build their business. One can hardly imagine this man having been the life of the party, running out onto the patio carrying his me in his arms, and jumping all dressed into the pool, yelling like Tarzan rescuing Jane. One can hardly imagine him playing in the ocean and throwing his kids high in the air. One can hardly imagine him as a loving grandfather playing Scrabble with his grandchildren. One can hardly imagine…
